# Precision Medicine: Genetic Repair of Retinitis Pigmentosa in Patient-Derived Stem Cells

**DOI:** 10.1038/srep19969

**Published:** 2016-01-27

**Authors:** Alexander G. Bassuk, Andrew Zheng, Yao Li, Stephen H. Tsang, Vinit B. Mahajan

**Affiliations:** 1 Department of Pediatrics, University of Iowa, Iowa City, IA, USA; 2 The Bernard & Shirlee Brown Glaucoma Laboratory, Departments of Ophthalmology, Pathology & Cell Biology, Institute of Human Nutrition, College of Physicians & Surgeons, Columbia University, New York, NY, USA; 3 Department of Ophthalmology and Visual Sciences, University of Iowa, Iowa City, IA, USA

## Abstract

Induced pluripotent stem cells (iPSCs) generated from patient fibroblasts could potentially be used as a source of autologous cells for transplantation in retinal disease. Patient-derived iPSCs, however, would still harbor disease-causing mutations. To generate healthy patient-derived cells, mutations might be repaired with new gene-editing technology based on the bacterial system of *clustered regularly interspersed short palindromic repeats* (CRISPR)/Cas9, thereby yielding grafts that require no patient immunosuppression. We tested whether CRISPR/Cas9 could be used in patient-specific iPSCs to precisely repair an *RPGR* point mutation that causes X-linked retinitis pigmentosa (XLRP). Fibroblasts cultured from a skin-punch biopsy of an XLRP patient were transduced to produce iPSCs carrying the patient’s c.3070G > T mutation. The iPSCs were transduced with CRISPR guide RNAs, Cas9 endonuclease, and a donor homology template. Despite the gene’s repetitive and GC-rich sequences, 13% of *RPGR* gene copies showed mutation correction and conversion to the wild-type allele. This is the first report using CRISPR to correct a pathogenic mutation in iPSCs derived from a patient with photoreceptor degeneration. This important proof-of-concept finding supports the development of personalized iPSC-based transplantation therapies for retinal disease.

Stem cell-derived cell transplantation in the eye is one therapy being explored for inherited retinal degenerations such as retinitis pigmentosa (RP). Recent clinical trials evaluating allogeneic retinal grafts derived from human embryonic stem cells (hESCs) show the procedure to be safe and potentially effective^1^. However, hESC-based treatments involve the controversial use of human embryos and pose a risk of immune-mediated rejection. Using a patient’s own cells for transplantation would avoid these pitfalls and is possible with induced pluripotent stem cells (iPSCs). Through established protocols^2^, fibroblasts from a skin biopsy can be returned to a pluripotent state and serve as a renewable, autologous source of replacement cells that avoids the ethical complications of hESCs.

Although the original disease-causing mutation would still be present in patient iPSCs, precise mutation correction is possible through gene editing techniques adapted from the bacterial *clustered regularly interspersed short palindromic repeats* (CRISPR)/Cas9 system^3^. The advantage of CRISPR is that its specificity depends largely on a guide RNA (gRNA) that can be readily programmed to target different genomic loci. In this study, we generated iPSCs from a patient with a mutation in the *retinitis pigmentosa GTPase regulator* (*RPGR*) gene, which causes an aggressive, X-linked variant of RP (XLRP). We tested whether CRISPR could precisely edit the pathogenic mutation and produce gene-corrected iPSCs for eventual use in autologous transplantation.

## Methods

All experimental protocols, including informed consent were apporved by the Institutional Review Board approval (AAAF1849) at Columbia University were obtained and all experiments were performed in accordance with relevant approved guidelines and regulations. Informed consent was obtained for all subjects. Autofluorescent imaging and optical coherence tomography were performed as described^4^. To generate iPSCs, dermal fibroblasts were cultured from the skin biopsy sample and transduced according to previously described protocols^5^. Pluripotency was demonstrated by teratoma assay conducted in immunodeficient mice (Charles River, Wilmington, MA) and immunocytochemistry staining for pluripotency markers.

CRISPR gRNAs were selected to minimize the off-target profile and to maximize the degree of homology with a target sequence directly centered on the mutation site. Candidates g58 and g59, (G58: GAAGTGGAAGGGGAGGTGGAAGG) and g59 (G59: AAGTGGAAGGGGAGGTGGAAGGG), were each inserted into the gRNA/Cas9 expression vector pBT-U6-Cas9-2A-GFP and transfected into a 293 cell line. Genomic DNA from test cells was amplified by PCR using primers for the *RPGR* region, and the PCR products were analyzed by the SURVEYOR® assay (Transgenomic, Omaha, NE) for DNA cleavage activity. The expression plasmid containing the g58 sequence and Cas9 were then transfected into the patient-derived C1145-11* iPSC line alongside a single-stranded donor oligonucleotide (ssODN) template. Ten days after transfection, cells were collected for PCR followed by targeted amplicon sequencing to assess for gene correction in the *open reading frame-15* (ORF15) region containing the mutation. Deep sequencing templates were obtained by PCR of the genomic sequence, which also detects some non-specific PCR products (not apparent on the gel) due to the very high deep sequencing coverage. These non-specific sequences were categorized under “non-specific/error”. Cells not transfected were used as controls.

## Results

**Figure 1:**
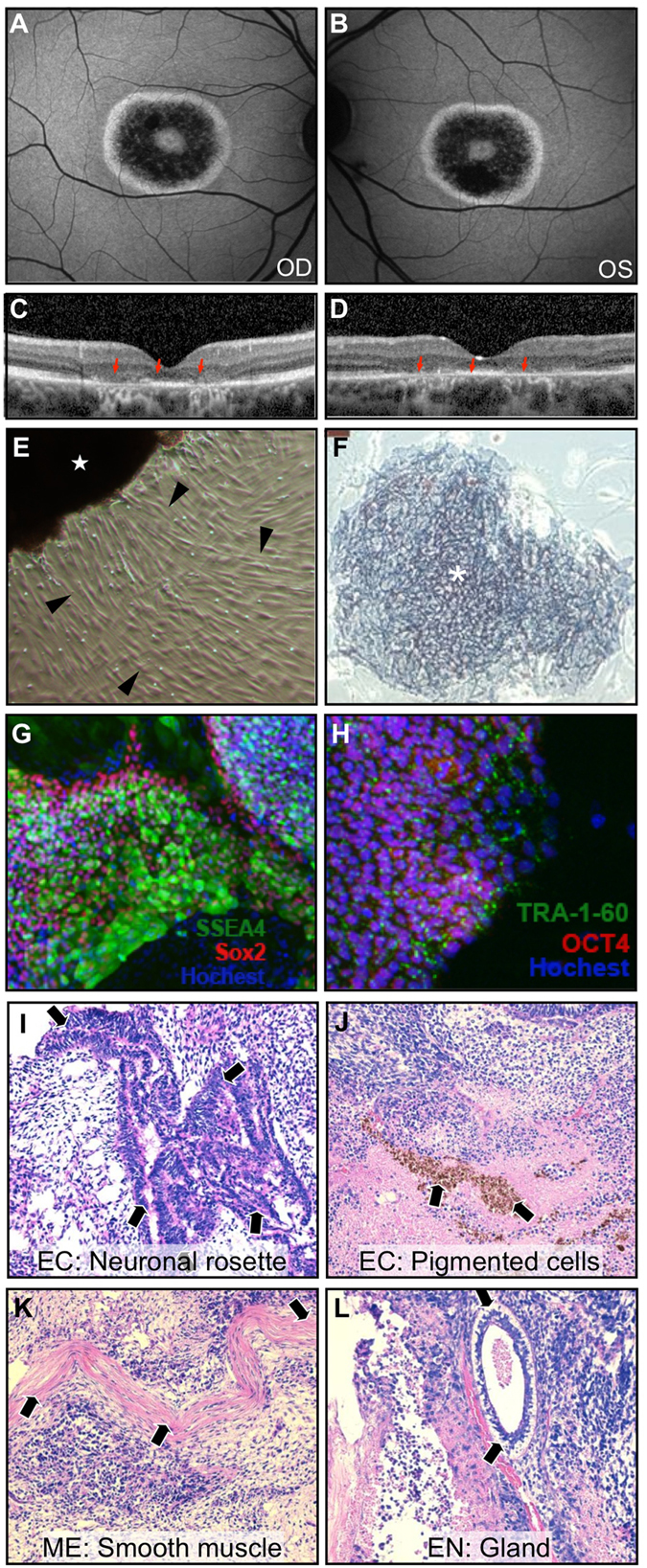
Clinical findings and iPSCs from an adult male XLRP patient. Autofluorescence imaging of the patient’s [**A**] right and [**B**] left eyes shows hyperautofluorescent bull’s-eye lesions in the macula corresponding to [**C**,**D**] loss of photoreceptor layers (red arrows) on optical coherence tomography. [**E**] A skin punch biopsy sample (star) was taken from the patient and used to culture fibroblasts (arrowheads) that were then transfected with transcription factors to yield efficient formation of [**F**] iPSC colonies (asterisk) that stain positively for alkaline phosphatase. With immunocytochemistry and fluorescence microscopy, expression of pluripotency markers [**G**] SSEA4, Sox2, and [**H**] TRA-1-60 and OCT4 is also detected. iPSCs injected into a severe combined immunodeficiency (SCID) mouse formed teratomas that were found on histology to contain cell types derived from all three germ layers: [**I**,**J**] neuronal rosettes and pigmented cells from the ectoderm (EC), [**K**] smooth muscle cells from the mesoderm (ME), and [**L**] gland cells from the endoderm (EN). The expression of pluripotency markers in the patient-derived cells and the ability to generate differentiated cells from all three primordial germ layers confirms that patient iPSCs are pluripotent.

We reported previously on the diagnosis of *RPGR*-associated XLRP in two brothers with significant vision loss^6^. Fundus examination and imaging showed a bull’s eye lesion with photoreceptor cell death near the fovea (Fig. 1A–D). Whole exome sequencing with Sanger sequencing confirmation uncovered a novel *RPGR* mutation (c.3070G > T, pGlu1024X) within the ORF15 exon^6^, a lengthy and repetitive, purine-rich region of the gene that accounts for >60% of all XLRP mutations^7^. We then cultured fibroblasts from a skin-punch biopsy and transformed them into patient-specific iPSCs. We confirmed that they were capable of differentiating into all three germ layers and expressed the pluripotency markers Oct-4, Sox2, SSEA-4, TRA-1-60, and alkaline phosphatase (Fig. 1E–L). In this state, they can be induced into retinal cells, but a critical limiting factor for transplantation is that they still carry a disease-causing mutation.

**Figure 2:**
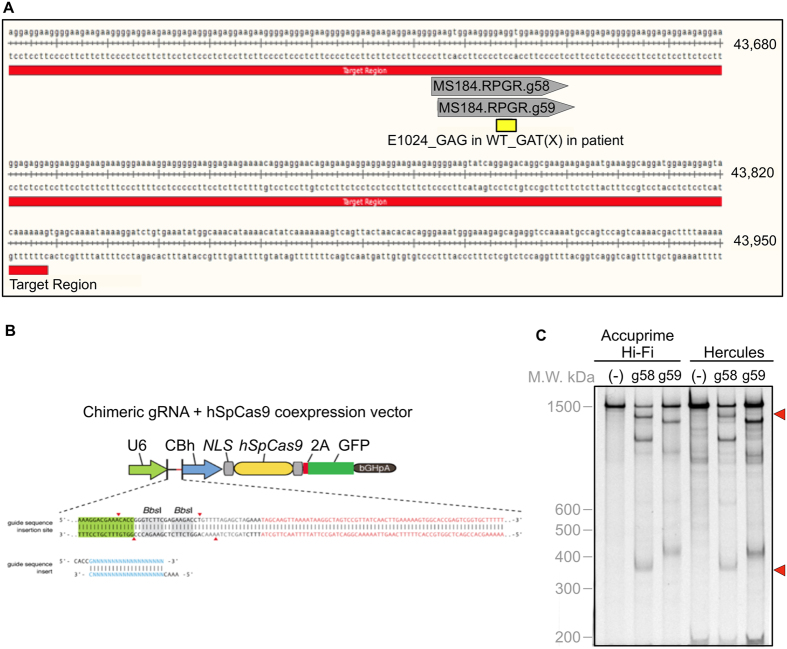
Validating gRNAs for gene targeting. [**A**] Two candidate gRNAs, g58 and g59, were selected based on their precise specificity for the mutation site within the ORF15 region of *RPGR*. [**B**] gRNAs were inserted into a combined expression vector alongside Cas9. The gRNA/Cas9 construct was transfected into HEK293 cells and the *RPGR* gene was amplified by PCR using both AccuPrime high fidelity *taq* polymerase as well as Herculase II polymerase (due to the GC-rich content of ORF15). [**C**] An *in vitro* SURVEYOR assay (Transgenomic, Omaha, NE) of the PCR products shows that g58 is more effective at directing cleavage by Cas9 (bands for the expected cleavage products are indicated by red arrowheads).

Next, we screened 21 gRNAs with high specificity to the region containing the patient’s *RPGR* mutation (see Supplementary Table). Two candidates were separately inserted into an expression vector also containing the Cas9 endonuclease responsible for mediating target DNA cleavage. A SURVEYOR® assay (Transgenomic, Omaha, NE) showed that one candidate, g58, had relatively high activity for the mutation site (Fig. 2). Because the *RPGR* sequence is highly repetitive, nonspecific bands were present (see methods). The calculation of cutting efficiency was based on the control band (before nuclease digestion) and the specific bands. The cutting efficiency was approximately 23% for g58. G59 was not active. Thus, g58 was used for gene editing.

**Figure 3:**
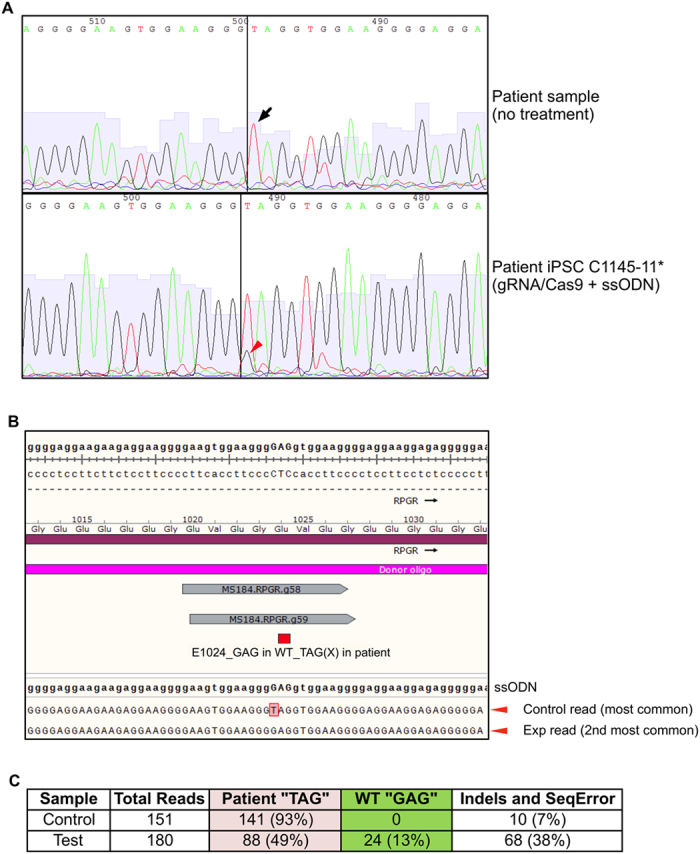
Correction of *RPGR* mutation in patient iPSC line. [**A**] Dideoxy sequencing of patient DNA (top panel) shows the “T” point mutation (black arrow). After gRNA/Cas9 and antisense single-stranded donor oligonucleotide (ssODN) were transfected into the patient-derived iPSC line (bottom panel), sequencing shows a minor “G” peak at the same position (red arrowhead), demonstrating correction of the G > T mutation in a fraction of IPSC. [**B**,**C**] Deep sequencing revealed 13% of sequences in treated cells had the correct “GAG” codon at the position of the patient’s original “TAG” stop codon point mutation. This correction was not observed when untransfected control cells were sequenced.

Following gRNA validation, we sought to determine whether Cas9-mediated cleavage at the *RPGR* mutation site could lead to homology-directed gene repair (HDR) in the presence of a co-transfected ssODN. We transfected patient iPSCs with the g58 gRNA/Cas9 expression plasmid and an *RPGR* anti-sense ssODN. Deep sequencing of transfected cells showed that 13% of reads contained precise correction of the mutation, with replacement of the premature stop codon “**T**AG” by the wild-type “**G**AG” codon encoding glutamate at residue 1024 (Fig. 3). Correction of mutation was not seen in any untransfected control iPSCs.

## Discussion

The goal of autologous cell replacement for retinal degenerative disease depends on the ability to correct a patient’s pathogenic mutation before transplantation. Here we have generated patient-specific iPSCs from an XLRP patient and showed that transfection of CRISPR gRNA/Cas9 alongside a donor homology template corrects a point mutation within the *RPGR* gene ORF15 region, a challenging DNA sequence to manipulate. We believe this is the first report of successful gene correction in human iPSCs associated with inherited retinal degeneration. The present study is significant because it demonstrates that gene editing can be engineered to precisely target a repeat*-*rich region with high GC content. *RPGR* spans a 59,000-bp region^8^ in a human genome containing over 3 billion bases, yet 13% of sequencing reads had correction of a single nucleotide mutation within the repetitive, low-complexity ORF15 region, demonstrating that despite presumed technical hurdles based on the genomic sequence, the *RPGR* gene is open to CRISPR repair. This rate of repair indicates that CRISPR gene editing may reasonably be part of an autologous transplantation treatment for other photoreceptor degenerations in the future.

The alternative, and currently the more developed, therapeutic modality for genetic retinal disease is gene supplementation therapy. Clinical trials for *RPE*-associated Leber congenital amaurosis reported in 2008 that gene therapy^9^ is safe and beneficial for patients. However, recently published three-year follow-up data found that initial gains in retinal sensitivity waned over time and were not accompanied by meaningful improvements in objective measures of visual function^10^. Moreover, gene therapy in animal models may produce much greater and more sustained rescue in humans at comparable vector doses, suggesting that interspecies genetic differences could limit translation to humans. These and other limits of gene therapy—such as its relative inability to treat dominantly inherited or late-stage disease—might be better addressed through a regenerative gene-corrected iPSC transplantation approach.

The 13% correction rate that we achieved is comparable to other studies of homology-directed CRISPR editing^11^, and exceeds previous iPSC gene editing studies reporting correction rates closer to 1%^12^. Such a rate can provide sufficient therapeutic cells, but gene-editing technology is also rapidly improving. The *piggyBac* transposon is a mobile genetic element that can insert and excise exogenous constructs in a “footprint free” manner for CRISPR in iPSCs^11^. Minimizing error-prone non-homologous end-joining at DNA cleavage sites through inhibition of DNA ligase IV can also increase the yield of repaired cells^13^. Definitive analysis of off-targeting rates will be required before eventual clinical use. With repaired patient-specific iPSCs in hand, the next step for iPSCs transplantation therapy is differentiating clonally corrected iPSCs into photoreceptors capable of integrating within the retina. Although a number of challenges remain, significant progress has been made in preclinical transplantation experiments using post-mitotic photoreceptor precursor cells or iPSC-derived e*n bloc* retinal tissue^14^,^15^.

## Conclusions

Here, we report that patient-derived iPSCs harboring a pathogenic RP mutation are amenable to precise correction by CRISPR/Cas9 gene editing. Our finding represents a concrete first step towards combining CRISPR/Cas9-mediated gene editing with autologous iPSCs to develop a personalized transplantation strategy adaptable to a wide array of retinal diseases.

## Additional Information

**How to cite this article**: Bassuk, A. G. *et al.* Precision Medicine: Genetic Repair of Retinitis Pigmentosa in Patient-Derived Stem Cells. *Sci. Rep.* **6**, 19969; doi: 10.1038/srep19969 (2016).

## Supplementary Material

Supplementary Table
