# Supplementary material for: Precision Medicine: Genetic Repair of Retinitis Pigmentosa in Patient-Derived Stem Cells
Source: Sci Rep. 2016 Jan 27;6:19969. doi: 10.1038/srep19969 (PMC4728485; doi:10.1038/srep19969)
Supplement: Supplementary Table [file srep19969-s1.pdf]

Supplementary Table 1. *RPGR* gRNAs.

Name	gRNA	long_0	long_1	long_2	short_0	SNP	SNP_count
**MS184.RPGR.g93**	CGAAGAAGAGAATGAAAGGCNGG	1	1	2	73	rs62636730:0.0163||rs111787313:0.0804	2
**MS184.RPGR.g91**	GAGGGGAAGTATCAGGAGACNGG	1	1	4	17	NA	0
**MS184.RPGR.g95**	GAATGAAAGGCAGGATGGAGNGG	1	1	7	159	rs62636730:0.0163	1
**MS184.RPGR.g82**	AGGGGGAAGGAGAAGAAAACNGG	1	1	12	106	NA	0
**MS184.RPGR.g84**	AAACAGGAGGAACAGAGAAGNGG	1	1	13	129	NA	0
**MS184.RPGR.g58**	GAAGTGGAAGGGGAGGTGGANGG	1	1	44	175	rs199896738:0.0387	1
**MS184.RPGR.g92**	CAGGCGAAGAAGAGAATGAANGG	1	2	6	128	rs62636730:0.0163||rs111787313:0.0804	2
**MS184.RPGR.g90**	GGAAGAAGAGGGGAAGTATCNGG	1	2	8	19	NA	0
**MS184.RPGR.g83**	GGGAAGGAGAAGAAAACAGGNGG	1	2	16	107	NA	0
**MS184.RPGR.g94**	GAAGAGAATGAAAGGCAGGANGG	1	2	21	87	rs62636730:0.0163	1
**MS184.RPGR.g59**	AAGTGGAAGGGGAGGTGGAANGG	1	2	38	148	rs199896738:0.0387	1
**MS184.RPGR.g85**	CAGGAGGAACAGAGAAGAGGNGG	1	3	40	150	NA	0
**MS184.RPGR.g78**	GGAGAAGAAAGGGAAAAGGANGG	1	4	90	248	NA	0
**MS184.RPGR.g86**	GAGGAACAGAGAAGAGGAGGNGG	1	5	38	438	NA	0
**MS184.RPGR.g79**	GAGAAGAAAGGGAAAAGGAGNGG	1	5	52	190	NA	0
**MS184.RPGR.g60**	AGTGGAAGGGGAGGTGGAAGNGG	1	5	73	302	rs199896738:0.0387	1
**MS184.RPGR.g65**	TGGAAGGGGAGGAAGGAGAGNGG	1	5	82	417	rs201134185:0.032	1
**MS184.RPGR.g74**	GAGAGGAGGAAGGAGAAGAANGG	1	6	98	328	rs200211905:0.02	1
**MS184.RPGR.g81**	GAAAGGGAAAAGGAGGGGGANGG	1	7	99	176	NA	0
**MS184.RPGR.g64**	GTGGAAGGGGAGGAAGGAGANGG	1	7	142	552	rs201134185:0.032	1
**MS184.RPGR.g76**	GGAAGGAGAAGAAAGGGAAANGG	1	8	106	333	NA	0

Precision Medicine: Genetic Repair of Retinitis Pigmentosa in Patient-Derived Stem Cells

Alexander G. Bassuk M.D, Ph.D., Andrew Zheng B.S., Yao Li M.D., Stephen H. Tsang M.D., Ph.D., Vinit B. Mahajan* M.D., Ph.D.
